# Progressive white matter changes following anterior temporal lobe resection for epilepsy^☆^

**DOI:** 10.1016/j.nicl.2013.12.004

**Published:** 2013-12-14

**Authors:** Gavin P. Winston, Jason Stretton, Meneka K. Sidhu, Mark R. Symms, John S. Duncan

**Affiliations:** Epilepsy Society MRI Unit, Department of Clinical and Experimental Epilepsy, UCL Institute of Neurology, Queen Square, London WC1N 3BG, England, UK

**Keywords:** Temporal lobe epilepsy, Anterior temporal lobe resection, Diffusion tensor imaging, White matter

## Abstract

Anterior temporal lobe resection (ATLR) is an effective treatment for refractory temporal lobe epilepsy (TLE). Widespread abnormalities in diffusion parameters involving the ipsilateral temporal lobe white matter and extending into extratemporal white matter have been shown in cross-sectional studies in TLE. However longitudinal changes following surgery have been less well addressed. We systematically assess diffusion changes in white matter in patients with TLE in comparison to controls before surgery and look at the longitudinal changes following ATLR at two timepoints (3–4 months, 12 months) using a whole brain approach.

We find predominantly unilateral baseline changes in temporal and extratemporal structures compatible with altered myelination (reduced fractional anisotropy, increased mean and radial diffusivity). Following surgery, these changes progress in efferent tracts from the resected temporal lobe compatible with Wallerian degeneration. However more superiorly in the corona radiata, internal and external capsules and nearby tracts, changes compatible with plasticity are observed (increased fractional anisotropy and axial diffusivity, reduced radial diffusivity).

There is little progression between 3–4 months and 12 months following surgery in patients with left TLE, but the changes become more widespread in patients with right TLE suggesting that plasticity occurs more slowly in this population. The neuropsychological correlates of such plasticity should be explored further.

## Highlights

• We study longitudinal changes in diffusion parameters after temporal lobe resection.
• Changes consistent with Wallerian degeneration are seen in nearby efferent tracts.
• More superiorly, diffusion changes compatible with plasticity are observed.
• Most of these changes are present by 3–4 months following left-sided surgery.
• Plasticity occurs more slowly after right-sided surgery with greater change by 1 year.

## 1 Introduction

Temporal lobe epilepsy (TLE)^1^ is the most common cause of refractory focal epilepsy with up to 40% of patients refractory to medication (Semah and Ryvlin, 2005). Anterior temporal lobe resection (ATLR) is an established and effective treatment (Wiebe et al., 2001). Cross-sectional white matter changes in TLE have been extensively studied but longitudinal changes following surgery have been less well addressed.

Diffusion tensor imaging (DTI) enables the non-invasive assessment of white matter structure (Basser, 1995) and is ideally suited for both cross-sectional and longitudinal studies. Widespread abnormalities in diffusion parameters have been demonstrated in patients with TLE not only involving the ipsilateral temporal lobe white matter but also extending into structures such as the fornix, cingulum, external capsule and corpus callosum (reviewed in (Gross, 2011)).

Several indices of tissue microstructure can be derived from DTI (reviewed in (Winston, 2012)). Following surgical axonal transection, Wallerian degeneration of downstream white matter tracts leads to a reduction in fractional anisotropy (FA), a measure of the degree of directionality of water diffusion and by inference tissue integrity, and an increase in mean diffusivity (MD), a measure of the magnitude of diffusion (Werring et al., 2000; Wieshmann et al., 1999). Wallerian degeneration comprises two phases with an acute phase of fragmentation and dying-back of axons (lasting days to weeks) followed by a chronic phase of degradation and phagocytosis of myelin sheaths (lasting weeks to months). These can be distinguished by considering two components of mean diffusivity, axial diffusivity (AD) along the length of the axon and radial diffusivity (RD) perpendicular to this. An initial decrease in AD representing axonal degeneration is followed by a later increase in RD representing the myelin loss in both animal models (Song et al., 2003) and in patients with epilepsy undergoing corpus callosotomy (Concha et al., 2006).

Diffusion parameters may also be altered by seizures themselves and a localized reduction in MD without alteration in FA may be observed immediately after a seizure (Diehl et al., 2005). Baseline alterations in diffusion parameters could either represent acute functional changes through fluid shifts induced by seizures or chronic structural changes (Concha et al., 2006). Following surgery, Wallerian degeneration would be expected to occur only in tracts transected during surgery. Therefore changes elsewhere, including contralateral cortex, could result from a reversal of the acute effects of seizures or structural plasticity, whereby chronic structural changes are reversed.

Although several studies have investigated changes in white matter structure following temporal lobe surgery (Concha et al., 2007; Faber et al., 2013; Liu et al., 2013; McDonald et al., 2010; Nguyen et al., 2011; Schoene-Bake et al., 2009; Yogarajah et al., 2010), key limitations include small group sizes, a lack of healthy controls or preoperative data for comparison, heterogeneity in the surgical approaches or the timing of the postoperative imaging and only studying a single postoperative timepoint, predefined regions of interest or a single diffusion parameter (Table 1).

The aim of the present study is to systematically assess the longitudinal changes in white matter following surgery avoiding these limitations. We investigate the baseline white matter changes in a large cohort of patients with TLE in comparison to healthy controls and then determine the longitudinal changes following a single surgical operation (ATLR) at two predefined postoperative timepoints using a whole brain approach. We employ tract-based spatial statistics (TBSS), a voxel-based technique optimized for diffusion data (Smith et al., 2006) that has high sensitivity to white matter changes in TLE (Focke et al., 2008) and avoids the problems of spatial smoothing (Jones et al., 2005) inherent in other techniques. This study design enables separation of the effects of the underlying disease from post-surgical changes, and a better understanding of the reversibility or otherwise of the baseline changes.

## 2 Methods

### 2.1 Subjects

We studied 20 patients with medically refractory left TLE (age range, 18–52 years; median, 35 years; 10 male) and 19 patients with right TLE (age range, 17–66 years; median, 41 years; 4 male) undergoing ATLR at the National Hospital for Neurology and Neurosurgery, London, United Kingdom. All patients had structural MRI scans performed at 3T, video electroencephalographic (EEG) telemetry, neuropsychology, neuropsychiatry, and if necessary intracranial EEG recordings prior to surgery (2 left, 1 right).

Diffusion tensor imaging (DTI) scans were acquired before surgery and at 3–4 months and 12 months following surgery. All patients underwent ATLR by a single surgeon who employed a modified Spencer approach. Access to the temporal horn of the lateral ventricle was from the floor of the middle cranial fossa via the collateral sulcus. An anterolateral resection was followed by en bloc resection of the mesial structures. In addition, 14 healthy age-matched controls without any history of neurological or psychiatric disease were studied with DTI scans at three similar timepoints. None of the subjects have been previously reported in longitudinal DTI studies.

The study was approved by the National Hospital for Neurology and Neurosurgery and the Institute of Neurology Joint Research Ethics Committee, and written informed consent was obtained from all subjects. Demographics and clinical data are listed in Table 2. There was no significant difference in the distribution of age, age of onset or duration of epilepsy between the groups (independent samples Kruskal–Wallis test). Postoperative seizure outcome was determined at 12 months using the ILAE classification (Wieser et al., 2001).

### 2.2 Magnetic resonance data

MRI studies were performed on a 3T GE Signa Excite HDx scanner (General Electric, Waukesha, Milwaukee, WI). Standard imaging gradients with a maximum strength of 40 mT m^− 1^ and slew rate 150 T m^− 1^ s^− 1^ were used. All data were acquired using a body coil for transmission, and 8-channel phased array coil for reception. DTI data were acquired using a cardiac-triggered single-shot spin-echo planar imaging sequence with TE of 73 ms. Sets of 60 contiguous 2.4 mm-thick axial slices were obtained covering the whole brain, with diffusion sensitizing gradients applied in each of 52 noncollinear directions (b value of 1200 s mm^− 2^ [δ = 21 ms, Δ = 29 ms, using full gradient strength of 40 mT m^− 1^]) along with 6 non-diffusion weighted scans. The gradient directions were calculated and ordered as described elsewhere (Cook et al., 2007). The field of view was 24 × 24 cm, and the acquisition matrix size was 96 × 96, zero filled to 128 × 128 during reconstruction, giving a reconstructed voxel size of 1.875 mm × 1.875 mm × 2.4 mm. The DTI acquisition time for a total of 3480 image slices was approximately 25 min (depending on subject heart rate).

### 2.3 DTI processing

Processing was undertaken with the FMRIB Diffusion Toolbox in FSL version 4.1.7 (Smith et al., 2004). Eddy current correction was performed using eddy_correct, brain extraction was performed with BET (Smith, 2002) and diffusion tensors were fitted using dtifit to give FA, MD, AD and RD images. Voxelwise statistical analysis of these images was carried out using TBSS (Smith et al., 2006).

For the standard TBSS protocol, all subjects' FA data was aligned to a common space using nonlinear registration (FNIRT) and the mean FA image was thinned to create a skeleton representing the centers of all tracts common to the group. Each subject's aligned FA data was projected onto this skeleton and the resulting data fed into voxelwise cross-subject statistics. The same transformations were used for the MD, RD and AD data.

Statistical inference was performed using a permutation based non-parametric method with 10,000 permutations (Nichols and Holmes, 2002) and threshold-free cluster enhancement (TFCE) was used to correct for multiple comparisons with a corrected p-value of < 0.05 considered significant. TFCE is more sensitive than traditional cluster-based methods and avoids the need to set an arbitrary threshold or to smooth data (Smith and Nichols, 2009). For the baseline comparisons, unpaired t-tests were used to determine the significant differences in diffusion parameters between either the left or right TLE group and healthy controls.

Significant clusters were displayed on the mean FA image from the group and identified with reference to the atlas tools supplied with FSL (ICBM-DTI-81 white-matter labels atlas and JHU white-matter tractography atlas). For both the cross sectional and longitudinal analyses, changes in FA and MD were the primary outcome with AD and RD measures used to clarify the nature of changes in significant regions.

### 2.4 Longitudinal data

For the longitudinal analysis, the standard TBSS protocol was adapted to account for the surgical resection and the non-independence of data. Preoperative images were registered to the FMRIB58_FA standard space template using the standard approach. The surgical resection was manually delineated by a single investigator (GPW) on the postoperative non-diffusion weighted image, dilated and used to create a brain mask excluding the resection. Each postoperative image was registered to the corresponding preoperative image using affine registration with FLIRT then non-linear registration with FNIRT with this mask used to weight the registration to avoid considering the area of resection. This warp and the warp from the preoperative image to the template were combined to directly register the postoperative image to the template with a single resampling and without considering the information from the resected area. This approach has been previously used by our group (Yogarajah et al., 2010).

For the statistical analysis, exchangeability blocks were defined each including the three datasets from each subject to account for the repeated measures and to ensure that the statistical test was of changes within subject. Comparisons were performed between the baseline scans and each set of postoperative scans considering each group (controls, left and right TLE) separately.

For each patient group (left or right TLE), a group resection mask was generated by registering the individual manually delineated resections to the template using the combined warp calculated as above, averaging these and then thresholding at 10%. The group resection masks were used to remove areas of significant change detected by TBSS due solely to an absence of that region following surgery.

After application of the group resection mask, clusters of significantly altered FA were deprojected and reverse normalized into the space of the native pre- and postoperative diffusion images. Mean FA, MD, AD and RD were calculated separately for clusters of decreased and increased FA at each timepoint to determine the magnitude of changes at the individual level. Baseline values and those at 12 months following surgery were compared using paired t-tests.

### 2.5 Role of the funding source

The sponsors had no role in study design; in the collection, analysis and interpretation of data; in the writing of the report; or in the decision to submit the article for publication.

## 3 Results

### 3.1 Baseline changes

**Supplementary Fig. 1:**
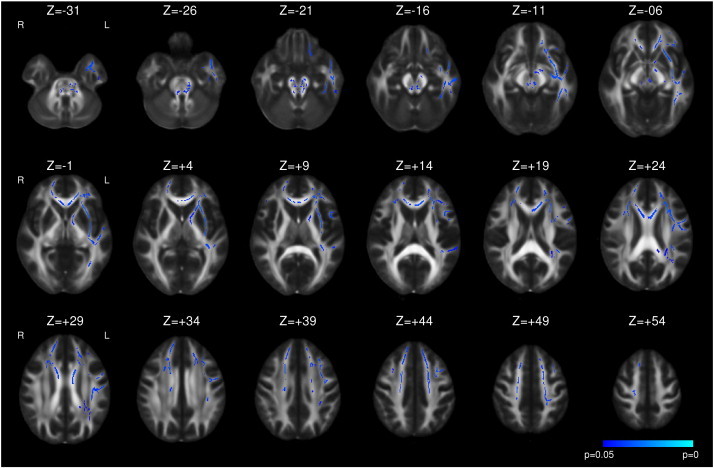
Whole brain TBSS analysis of changes in FA in left TLE compared to healthy controls. Decreases (blue to light blue) are shown with threshold-free cluster-enhanced correction (p < 0.05).

**Supplementary Fig. 2:**
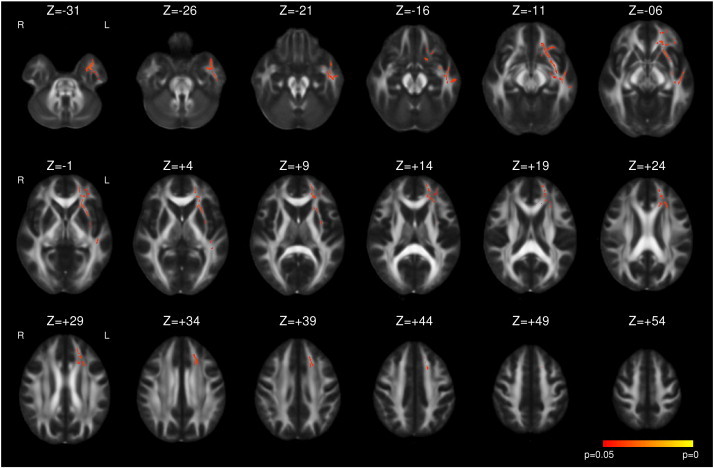
Whole brain TBSS analysis of changes in MD in left TLE compared to healthy controls. Increases (red to yellow) are shown with threshold-free cluster-enhanced correction (p < 0.05).

In patients with left TLE, there were widespread reductions in FA compared to controls. Affected areas included the ipsilateral fornix, internal and external capsules, uncinate fasciculus (UF), superior longitudinal fasciculus (SLF), inferior longitudinal fasciculus (ILF), inferior fronto-occipital fasciculus (IFOF) and the posterior thalamic radiation/optic radiation (PTR/OR) (Supplementary Fig. 1, Supplementary Table 1). There were also bilateral reductions in FA within the corpus callosum, cerebral peduncles and corona radiata. More limited increases in MD were observed in the left hemisphere including the fornix, internal and external capsules, UF, ILF, IFOF and corpus callosum (Supplementary Fig. 2, Supplementary Table 1).

In patients with left TLE, there were widespread reductions in FA compared to controls. Affected areas included the ipsilateral fornix, internal and external capsules, uncinate fasciculus (UF), superior longitudinal fasciculus (SLF), inferior longitudinal fasciculus (ILF), inferior fronto-occipital fasciculus (IFOF) and the posterior thalamic radiation/optic radiation (PTR/OR) (Supplementary Fig. 1, Supplementary Table 1). There were also bilateral reductions in FA within the corpus callosum, cerebral peduncles and corona radiata. More limited increases in MD were observed in the left hemisphere including the fornix, internal and external capsules, UF, ILF, IFOF and corpus callosum (Supplementary Fig. 2, Supplementary Table 1).

**Supplementary Fig. 3:**
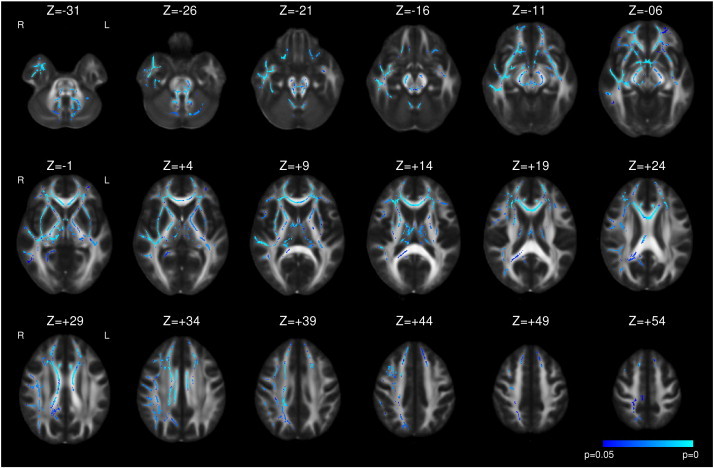
Whole brain TBSS analysis of changes in FA in right TLE compared to healthy controls. Decreases (blue to light blue) are shown with threshold-free cluster-enhanced correction (p < 0.05).

**Supplementary Fig. 4:**
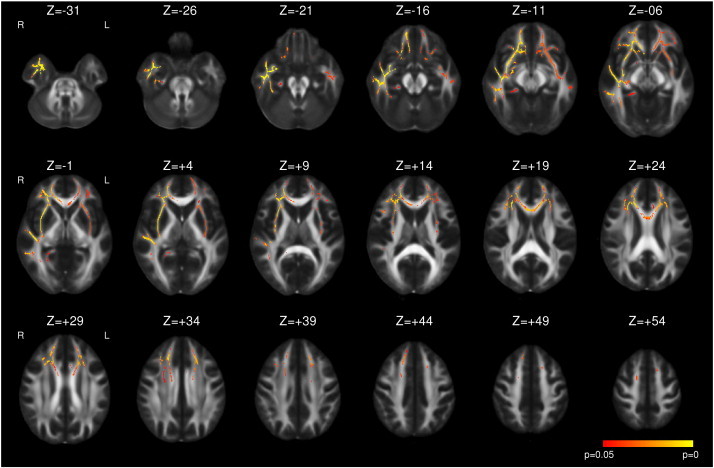
Whole brain TBSS analysis of changes in MD in right TLE compared to healthy controls. Increases (red to yellow) are shown with threshold-free cluster-enhanced correction (p < 0.05).

In patients with right TLE, there were also widespread reductions in FA compared in controls. These were predominantly bilateral involving the fornix (both crura and column/body), internal and external capsules, UF, ILF, IFOF, PTR/OR, cingulate gyrus, corpus callosum, cerebral peduncles and corona radiata. In addition, there were reductions in the right parahippocampal cingulum (PHC) and SLF (Supplementary Fig. 3, Supplementary Table 2). Bilateral increases in MD were observed in the internal and external capsules, UF, IFOF, corpus callosum and cerebral peduncles whilst increases confined to the right hemisphere were seen in the fornix, PHC, SLF, ILF and PTR/OR (Supplementary Fig. 4, Supplementary Table 2).

In patients with right TLE, there were also widespread reductions in FA compared in controls. These were predominantly bilateral involving the fornix (both crura and column/body), internal and external capsules, UF, ILF, IFOF, PTR/OR, cingulate gyrus, corpus callosum, cerebral peduncles and corona radiata. In addition, there were reductions in the right parahippocampal cingulum (PHC) and SLF (Supplementary Fig. 3, Supplementary Table 2). Bilateral increases in MD were observed in the internal and external capsules, UF, IFOF, corpus callosum and cerebral peduncles whilst increases confined to the right hemisphere were seen in the fornix, PHC, SLF, ILF and PTR/OR (Supplementary Fig. 4, Supplementary Table 2).

In both groups, an increase in RD without a change in AD was found to underlie the observed changes in FA and MD. There were no significant changes in AD and no increases in FA or reductions in MD in either group.

### 3.2 Longitudinal changes

**Fig. 1:**
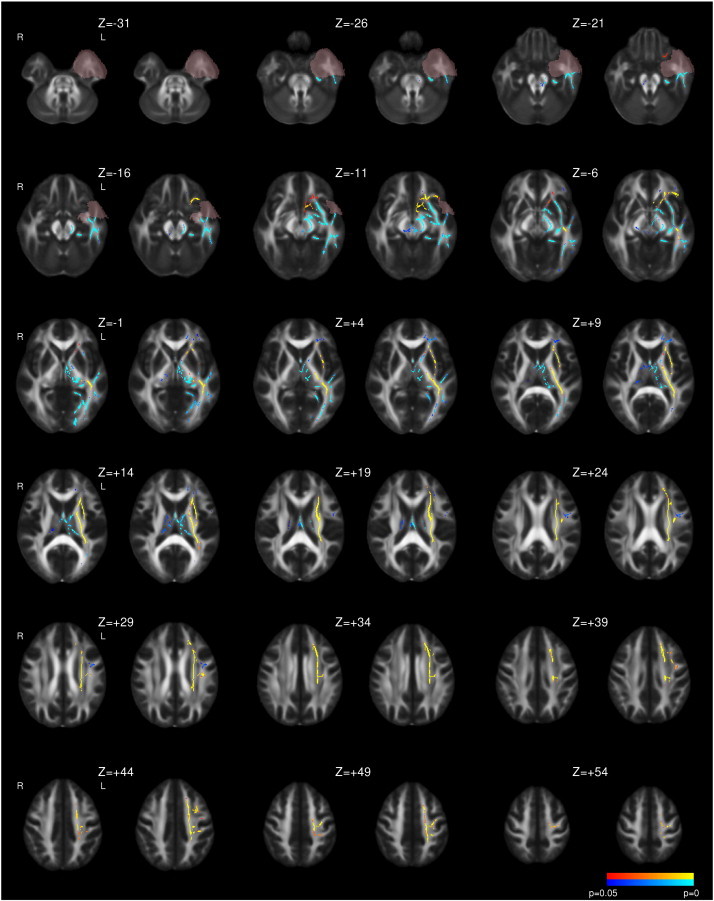
Whole brain TBSS analysis of changes in FA following left ATLR. Decreases (blue to light blue) and increases (red to yellow) are shown with threshold-free cluster-enhanced correction (p < 0.05). For each pair of images, the comparison of 3–4 months following surgery to preoperative imaging is on the left and the comparison of 12 months following surgery to preoperative imaging is on the right. The mask for the resected area is shown in pink.

**Fig. 2:**
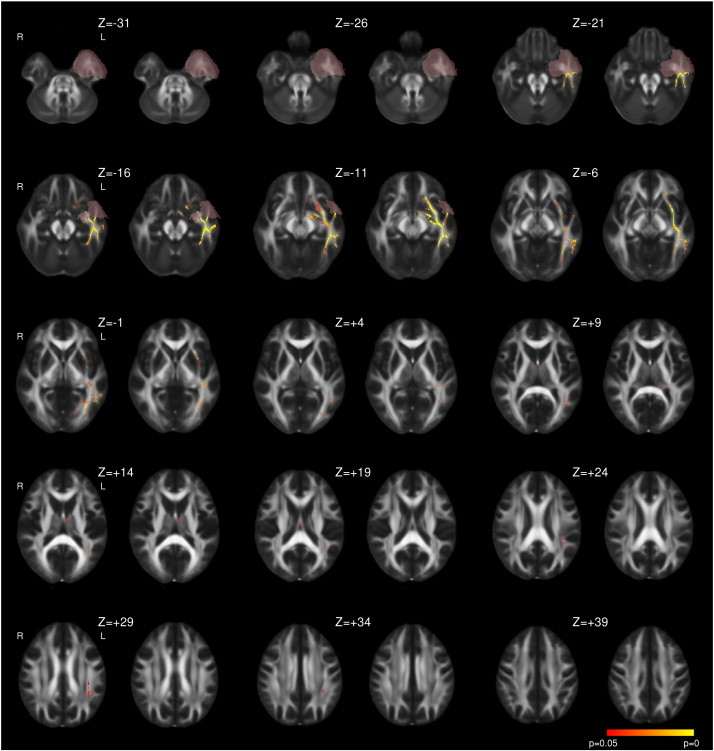
Whole brain TBSS analysis of changes in MD following left ATLR. Same conventions as Fig. 1.

Following left ATLR, a reduction in FA was seen in efferent tracts from the temporal lobe including the fornix, UF, PHC and PTR/OR (Fig. 1 and Table 3). Decreases in FA were observed in the more inferior parts of other tracts, including the internal and external capsules, corona radiata, SLF, ILF, IFOF, whilst a large contiguous area of increased FA was seen in the more superior parts of these tracts. Changes were present by 3–4 months with only a small further increase in extent of both decreased and increased FA by 12 months (Fig. 1). A more limited region of increased MD was seen at 3–4 months including the fornix, UF, internal and external capsules, corona radiata, PTR/OR, SLF, ILF and IFOF that became less extensive by 12 months (Fig. 2).

**Fig. 3:**
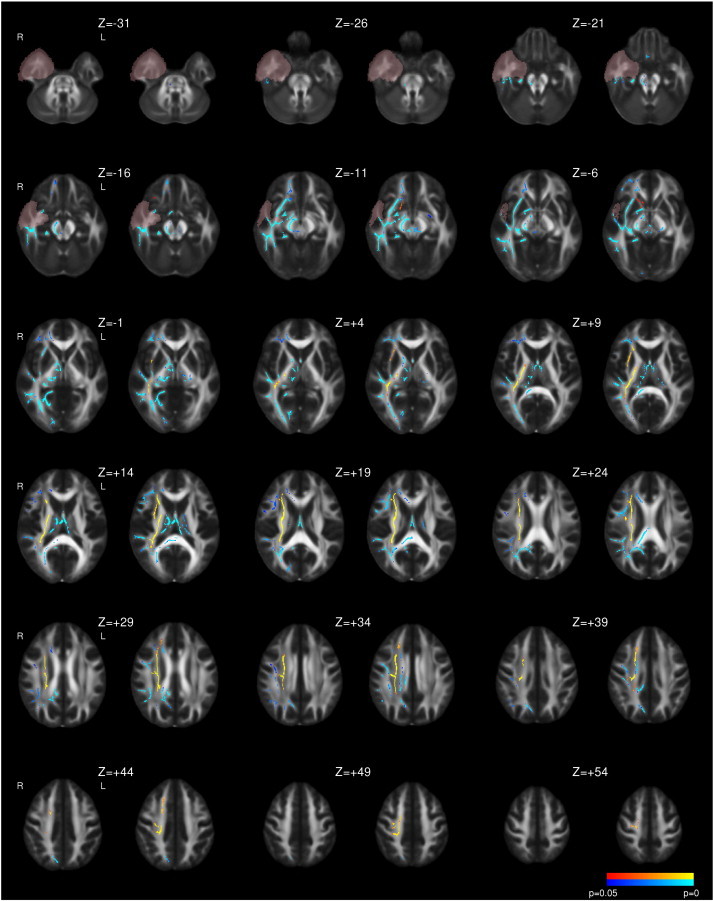
Whole brain TBSS analysis of changes in FA following right ATLR. Same conventions as Fig. 1.

**Fig. 4:**
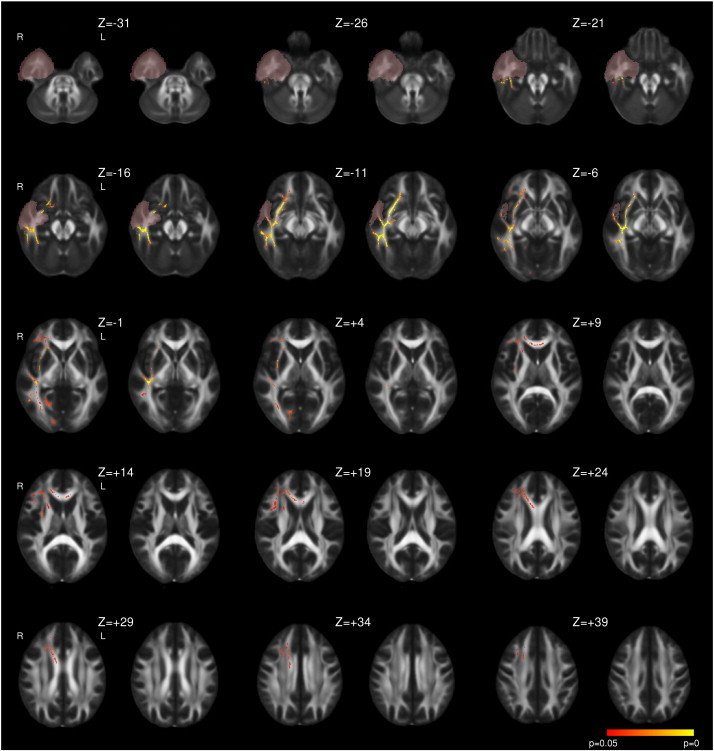
Whole brain TBSS analysis of changes in MD following right ATLR. Same conventions as Fig. 1.

Following right ATLR, a reduction in FA was seen in the corpus callosum and efferent tracts from the temporal lobe including the fornix, UF and PHC/cingulum (Fig. 3 and Table 4). Decreases in FA were also observed in the more inferior parts of the internal and external capsules, corona radiata, PTR/OR, SLF, ILF and IFOF whilst there were increases in a contiguous region including the more superior parts of these structures. In contrast to left TLE, whilst the majority of changes were present by 3–4 months, these changes became more widespread by 12 months and only reached significance at the later timepoint in regions such as the right external capsule (Fig. 3). Less widespread increases in MD were observed including the fornix, corpus callosum, internal and external capsules, corona radiata, SLF, ILF, IFOF and UF. Many of these changes were only significant at 3–4 months having resolved by 12 months (Fig. 4).

In both groups of patients, the areas of increased MD were mostly a subset of those with decreased FA and the changes in these regions were predominantly driven by an increase in RD. There were some changes in AD, with reductions in the cerebral peduncles, ipsilateral fornix and part of the internal capsule and some increases in regions of increased MD.

In the deprojected reverse normalized cluster of decreased FA, the decrease in FA of 10.1% (left TLE, p < 0.001) or 7.5% (right TLE, p < 0.001) was associated with an increase in MD by 5.8% (left, p < 0.001) or 3.3% (right, p < 0.001) and RD by 10.9% (left, p < 0.001) or 7.3% (right, p < 0.001) but no significant change in AD (Table 5). In contrast, in the contiguous area of increased FA and stable MD, the predominant change was an increase in AD with smaller areas of reduced RD in the ipsilateral internal capsule, corona radiata and posterior thalamic radiation. The increase in FA was 8.5% (left, p < 0.001) or 10.3% (right, p < 0.001), the increase in AD was 4.3% (left, p < 0.001) or 5.8% (right, p < 0.001) and the decrease in RD was 3.8% (left, p < 0.001) or 5.3% (right, p < 0.001) with no significant change in MD (Table 5).

No changes in any diffusion parameter were observed in healthy controls at either timepoint.

## 4 Discussion

### 4.1 Summary of findings

At baseline, widespread reductions in FA encompassing both temporal and extratemporal structures were seen with more limited areas of increased MD. The changes were predominantly unilateral in left TLE but more bilateral in right TLE. Increased RD was found to underlie the change.

Following surgery, decreases in FA were seen in ipsilateral efferent tracts from the temporal lobe including the fornix, UF and cingulum. Elsewhere in the internal and external capsules, corona radiata, SLF, ILF and IFOF, ipsilateral decreases in FA were seen in the more inferior portions whilst increases in FA were seen in the more superior portions. The areas of reduced FA that were partly accompanied by increased MD were associated mainly with an increase in RD whilst the areas of increased FA but stable MD were associated with an increase in AD and to a lesser extent a reduction in RD.

In left TLE, little progression in FA changes was observed between 3–4 months and 12 months following surgery whilst in right TLE, the changes became more marked over this period. Changes in MD were less extensive by 12 months, particularly in right TLE. In both groups, postoperative changes were predominantly ipsilateral with only minimal contralateral changes.

### 4.2 Biological basis of baseline changes

The baseline changes of reduced FA, increased MD and RD are in keeping with previous reports (Arfanakis et al., 2002; Concha et al., 2005; Focke et al., 2008; Gross et al., 2006) and are likely to be a consequence of damaged or deficient myelination (Song et al., 2003). Other possibilities include altered membrane permeability or altered neuronal packing density (Arfanakis et al., 2002). We found more bilateral changes in right TLE than left TLE, the converse of previous studies (Ahmadi et al., 2009; Kemmotsu et al., 2011). However as diffusion changes in right TLE were found to be more widespread and bilateral in females than males (Oguz et al., 2013), the gender imbalance in our right TLE group, which was 79% female, could explain this difference. The presence of baseline changes highlights the importance of including controls and preoperative imaging, as a previous study comparing postoperative imaging in patients to healthy controls and demonstrating widespread reductions in FA within ipsilateral tracts was unable to conclude whether these changes were pre-existing as a result of the disease or developed following surgery (Schoene-Bake et al., 2009).

### 4.3 Dichotomy of longitudinal changes

Following surgery, the observed changes can be split into two contrasting regions. In tracts involved in or nearby the resection, the observed changes (reduced FA, increased MD and RD) are in keeping with the chronic phase of Wallerian degeneration following axonal transaction and comparable to previously reported progressive changes following surgery within the ipsilateral fornix and cingulum (Concha et al., 2007) and other tracts (McDonald et al., 2010; Yogarajah et al., 2010).

There is a second distinct more superiorly located region including the corona radiata, internal and external capsules and nearby tracts in which an increase in FA without altered MD is driven mainly by a rise in AD but also a fall in RD. Our group has previously reported similar longitudinal changes in a different cohort (Yogarajah et al., 2010) which was the first study to conclusively report an increase in FA following surgery. Including a control group in the present study allows a comparison of these changes to baseline abnormalities and by studying two postoperative timepoints we demonstrated that in right TLE, additional changes develop by 12 months, particularly an increase in FA in the external capsule.

### 4.4 Biological implications and causes of increased postoperative FA

Postoperative changes could represent reversal of a functional, metabolic, disruption (Concha et al., 2005; Yogarajah et al., 2010) and support for this view comes from other imaging modalities. FDG-PET shows a normalization of glucose metabolism in the remaining ipsilateral temporal cortex (Gogoleva et al., 1997) and extratemporal regions including the inferior frontal lobe, thalamus and parietal lobe (Spanaki et al., 2000; Takaya et al., 2009) following surgery. Moreover, magnetic resonance spectroscopy shows that N-acetylaspartate (NAA)/creatine (Cr) ratios normalize in the ipsilateral (Cendes et al., 1997; Serles et al., 2001) and contralateral (Hugg et al., 1996; Lantz et al., 2006) temporal cortex in patients rendered seizure free by surgery.

Following a seizure, an acute reduction in MD without a change in FA is observed (Diehl et al., 2005). This differs from the baseline changes (reduced FA, increased MD), is not the converse of the recovery following surgery (increased FA, unchanged MD) and the acute effects of seizures are unlikely to have a significant contribution in our study as all but three patients had been seizure free for the 24 h prior to baseline imaging.

An alternative explanation is underlying structural changes that may partially reverse. Previous data suggest that the bilateral baseline diffusion changes within the fornix, cingulum and external capsule fail to normalize in the contralateral hemisphere following surgery that resulted in seizure freedom (Concha et al., 2007) and the authors used these data to argue for an underlying preoperative structural, rather than functional change. In the present study, we show that some of the preoperative diffusion changes do reverse in more distant ipsilateral cortex, although similarly we did not see any resolution of contralateral changes. Changes could be driven by the resolution of structural changes or the effects of plasticity and neuronal remodeling. For example, the increase in AD could represent axonal regeneration whilst the reduction in RD could represent improvement in myelination.

Caution must however be attached to the interpretation of the observed postoperative increases in FA as selective degeneration of a single fiber population from two crossing fiber populations in the corona radiata has previously been shown to lead to a paradoxical rise in FA in patients with Alzheimer's disease (Douaud et al., 2011). Whilst this may be contributory in the corona radiata and adjacent tracts, it seems less likely in the internal and external capsules where fibers are expected to be more coherent.

### 4.5 Time course of postoperative change

The time course of postoperative changes can only be assessed by longitudinal studies involving more than one postoperative timepoint. McDonald and colleagues looked at changes in FA using a region-of-interest based approach in 7 patients at 2 months and 12 months following ATLR (McDonald et al., 2010). A reduction of FA in ipsilateral tracts including the UF, PHC, ILF, IFOF, corpus callosum and bilateral fornix was seen by 2 months with no further change by 12 months. These changes were confirmed in the present study, but by using a larger sample and whole brain analysis we were also able to detect increases in FA and show some limited progression between 3–4 months and 12 months. We also investigated other diffusion indices to clarify the nature of these changes.

Another study looked at patients with left TLE only undergoing SAH (Faber et al., 2013). Follow-up was at 3–6 months and 12 months, although the intervals were wide with the late group (11–23 months). The reported changes were reduced FA in the fornix and PHC at 3–6 months and later in the UF. Supplementary data suggest that reductions in FA were more widespread predominantly ipsilaterally along with some increases in the ipsilateral SLF and ILF, which is in keeping with the present findings. However a serious limitation of this study is that the groups at 3–6 months and 12 months were not the same thus precluding any true inference on longitudinal changes.

The time course we observed suggests that the majority of postoperative change occurs within the first few months following surgery, although changes in right TLE appeared to occur slightly later and continue beyond this point. No previous studies exist to compare the time course between left and right TLE. The majority of our patients were right handed and structural plasticity may occur more readily in the dominant hemisphere. Alternatively, the more bilateral baseline changes in our right TLE group may contribute to the slower time course of plasticity. Since our groups were matched on demographic and clinical variables aside from gender, this finding needs replication to ensure that gender is not a contributor.

### 4.6 Strengths and limitations

This is the first study of patients undergoing temporal lobe epilepsy surgery with diffusion imaging both before and at two postoperative timepoints that also includes a control group with imaging at the same timepoints. This enables a baseline comparison and a detailed study of longitudinal changes. The large cohort allowed separate analysis of left and right TLE. All patients underwent the same surgery (ATLR) by the same surgeon, as other operations may yield different consequences.

Rather than limiting the analysis to certain tracts of interest, a whole brain analysis was performed using TBSS to give the highest sensitivity and reliability and hand drawn resection masks were employed to optimize image registration and exclude the resected regions from the detected changes. Whilst some prior studies investigated only changes in FA, we considered multiple diffusion parameters to provide a clearer explanation for the changes.

As the majority of changes were present at the first postoperative scan, further information may come from earlier timepoints. Without these, the early stages of Wallerian degeneration cannot be detected. A recent paper looking at diffusion changes in the first week following surgery found findings inconsistent with axonal injury models but that could instead have resulted from axonal swelling (Liu et al., 2013). There are also practical difficulties in early scans. Future work should look at changes at earlier timepoints such as 2, 4 and 8 weeks.

We included all patients, both seizure free and otherwise, in the analysis whilst others have only studied seizure free patients (Concha et al., 2007; McDonald et al., 2010; Nguyen et al., 2011). Although the majority of patients were seizure free, in patients with ongoing seizures this could induce diffusion changes that confound any changes from plasticity and reduce the sensitivity of the analysis.

## 5 Conclusions

We demonstrated widespread baseline changes in diffusion parameters in patients with TLE in the temporal lobe and extratemporal structures. Following surgery, changes consistent with Wallerian degeneration occurred in the ipsilateral temporal lobe and adjacent tracts whilst there are changes compatible with structural plasticity more superiorly in the ipsilateral hemisphere. The majority of changes were present within 3–4 months after surgery, but further progression occurred up to 12 months particularly in right TLE. Only minimal change occurred in the contralateral cortex following surgery. This study provides a description of the postoperative changes and future work will look at how these changes relate to changes in clinical outcomes, such as language or memory function on neuropsychological assessment.

The following are the supplementary data related to this article.

Supplementary tables.

Supplementary data to this article can be found online at http://dx.doi.org/10.1016/j.nicl.2013.12.004.

## Figures and Tables

**Table 1:** Previous studies on longitudinal diffusion changes following temporal lobe surgery. ATLR = anterior temporal lobe resection, HS = hippocampal sclerosis, ROI = region-of-interest analysis, SAH = selective amygdalo-hippocampectomy. Abbreviations for tracts as per text.

Paper	Cohort (laterality)	Surgery (imaging timepoints)	Analysis	Findings
Concha et al. (2007)	8 HS (6L, 2R) 22 controls	ATLR/SAH (before, 1 year after)	Tractography of fornix, cingulum ROI of EC, CC	Baseline reduced FA, increased MD/RD in bilateral fornix, cingulum, EC Progressive ipsilateral changes but no contralateral normalization after surgery
Schoene-Bake et al. (2009)	40 HS (19L, 21R) 28 controls	ATLR/SAH (3–11 years after only)	Whole brain (TBSS)	Reduced FA in predominantly ipsilateral tracts including cingulum, SLF, ILF, IFOF, corpus callosum
McDonald et al. (2010)	7 HS (3L, 4R) No controls	ATLR (before, 2 months and 12 months after)	ROI of key tracts	Reduced FA in bilateral fornix, ipsilateral UF, PHC, ILF, IFOF, CC by 2 months, no further change at 12 months
Yogarajah et al. (2010)	46 TLE (26L, 20R) No controls	ATLR (before, 4 months after)	Whole brain (TBSS)	LHS: reduced FA ipsilateral UF, PHG, SLF, OR, bilateral fornix, ILF; increased FA IC, EC, corona radiata; increased MD ipsilateral UF, EC, ILF RHS: reduced FA ipsilateral UF, PHG, SLF, IFOF, OR, bilateral fornix, ILF; increased FA corona radiata; increased MD ipsilateral UF, EC, bilateral fornix
Nguyen et al. (2011)	22 HS (11L, 11R) Only 10 post-op No controls	ATLR (before, 2–7 months after)	Whole brain (TBSS) Images flipped to combine analysis	Baseline asymmetry in FA within hippocampus, fornix, UF, corpus callosum (no controls for comparison) Postoperative increased MD in ipsilateral anterior temporal region but no change in FA
Faber et al. (2013)	20 TLE (left) No controls	SAH (before, 3–6 months and 12 months after)	Whole brain (TBSS)	Reduced FA in left cingulum, fornix at early timepoint and subsequently reduced FA in left UF
Liu et al. (2013)	6 TLE (3L, 3R) 3 controls	ATLR/SAH (before, multiple early scans)	Tractography of fornix	Ipsilateral reduction in MD/AD/RD by 2 days, reduction in FA and rise in MD/RD by 1–4 months

**Table 2:** Clinical and demographic characteristics of patients and healthy controls. Data are given as range (median). HS = hippocampal sclerosis, DNET = dysembryoplastic neuroepithelial tumor, EFS = end folium sclerosis, FCD = focal cortical dysplasia.

Group	Controls (n = 14)	LTLE (n = 20)	RTLE (n = 19)
Gender (M/F)	8/6	10/10	4/15
Handedness (R/L)	10/4	17/3	17/3
Age at scan	22–53 (39.5)	18–52 (35)	17–66 (41)
Age at onset	N/A	0.3–35 (12)	1–44 (11)
Duration of epilepsy	N/A	2–51 (16)	3–52 (22)
Days to first postoperative scan	75–361 (168)	81–183 (105.5)	75–243 (103)
Days to second postoperative scan	217–944 (456)	284–473 (377.5)	337–445 (384)
ILAE outcome at 12 months	N/A	Group 1: 15 Group 2: 3 Group 3: 1 Group 4: 1	Group 1: 14 Group 2: 1 Group 3: 2 Group 4: 1 Group 5: 1
Histological diagnosis	N/A	HS (n = 13) HS + DNET (n = 2) EFS (n = 1) Gliosis (n = 1) Cavernoma (n = 2) Ependymoma (n = 1)	HS (n = 11) HS + FCD (n = 1) EFS (n = 4) DNET (n = 3)

**Table 3:** Summary of longitudinal diffusion changes following surgery in whole-brain analysis of left TLE patients. The p-values for the maximum change at 3–4 months and 12 months within each region are given. A single p-value is quoted if they are the same.

Postoperative reduction in FA following left ATLR	Left fornix (cres) (p < 0.001) Column/body of fornix (p < 0.001) Left uncinate fasciculus (p < 0.001) Left parahippocampal cingulum (p < 0.001) Left cerebral peduncle (p < 0.001) (containing corticopontine, corticospinal, corticobulbar fibers) Left posterior thalamic radiation/optic radiation (p < 0.001, p = 0.009) Left corticospinal tract (p < 0.001, p = 0.009) Left superior cerebellar peduncle (p = 0.034 at 3–4 months only) Right cerebral peduncle (p = 0.034 at 12 months only)
Postoperative reduction in FA following left ATLR only in part of tract (increases elsewhere)	Left anterior limb of internal capsule (p < 0.001, p = 0.002) Left posterior limb of internal capsule (p < 0.001) Left retrolenticular portion of internal capsule (p = 0.005 at 12 months only) Left anterior corona radiata (p = 0.034, p = 0.017) Left inferior fronto-occipital fasciculus (p < 0.001) Left inferior longitudinal fasciculus (p < 0.001) Left external capsule (p < 0.001) Left superior longitudinal fasciculus (p < 0.001) (temporal portion)
Postoperative increases in FA following left ATLR	Left anterior limb of internal capsule (p < 0.001) Left posterior limb of internal capsule (p < 0.001) Left retrolenticular portion of internal capsule (p < 0.001) Left anterior corona radiata (p < 0.001) Left superior corona radiata (p < 0.001) Left posterior corona radiata (p < 0.001) Left posterior thalamic radiation/optic radiation (p < 0.001) Left inferior longitudinal fasciculus (p < 0.001) Left inferior fronto-occipital fasciculus (p < 0.001) Left external capsule (p < 0.001) Left superior longitudinal fasciculus (p < 0.001) (frontoparietal portion)
Postoperative increases in MD following left ATLR	Left retrolenticular portion of internal capsule (p = 0.024, p = 0.002) Left anterior corona radiata (p = 0.004 at 12 months only) Left posterior thalamic radiation/optic radiation (p = 0.008, p = 0.019) Left inferior fronto-occipital fasciculus (p = 0.003, p < 0.001) Left inferior longitudinal fasciculus (p = 0.003, p < 0.001) Left external capsule (p = 0.002, p < 0.001) Left fornix (p = 0.003, p < 0.001) Left superior longitudinal fasciculus (p = 0.025 at 3–4 months only) Left uncinate fasciculus (p = 0.002, p < 0.001)

**Table 4:** Summary of longitudinal diffusion changes following surgery in whole-brain analysis of right TLE patients. The p-values for the maximum change at 3–4 months and 12 months within each region are given. A single p-value is quoted if they are the same.

Postoperative reduction in FA following right ATLR	Corpus callosum, genu (p = 0.035, p = 0.014) Corpus callosum, body (p = 0.035, p = 0.004) Corpus callosum, splenium (p = 0.001, p = 0.003) Right fornix (cres) (p < 0.001) Column/body of fornix (p < 0.001) Right cingulate gyrus (p = 0.003) Right parahippocampal cingulum (p < 0.001) Right uncinate fasciculus (p < 0.001) Right cerebral peduncle (p < 0.001) Left cerebral peduncle (p = 0.011, 12 months only) Left anterior limb of internal capsule (p = 0.012, 12 months only) Right corticospinal tract (p = 0.002, 12 months only) Right superior cerebellar peduncle (p = 0.039, 12 months only) Middle cerebellar peduncle (p = 0.004, 12 months only)
Postoperative reduction in FA following right ATLR only in part of tract (increases elsewhere)	Right anterior limb of internal capsule (p < 0.001, p = 0.003) Right posterior limb of internal capsule (p < 0.001) Right retrolenticular portion of internal capsule (p = 0.001, 12 months only) Right anterior corona radiata (p < 0.001) Right superior corona radiata (p = 0.011, 12 months only) Right posterior corona radiata (p = 0.003, p = 0.004) Right posterior thalamic radiation/optic radiation (p < 0.001, p = 0.003) Right inferior fronto-occipital fasciculus (p < 0.001) Right inferior longitudinal fasciculus (p < 0.001) Right external capsule (p < 0.001) Right superior longitudinal fasciculus (p < 0.001)
Postoperative increases in FA following right ATLR	Right anterior limb of internal capsule (p < 0.001) Right posterior limb of internal capsule (p < 0.001) Right retrolenticular portion of internal capsule (p < 0.001) Right anterior corona radiata (p < 0.001) Right superior corona radiata (p < 0.001) Right posterior corona radiata (p < 0.001) Right posterior thalamic radiation/optic radiation (p < 0.001) Right inferior fronto-occipital fasciculus (p < 0.001) Right inferior longitudinal fasciculus (p = 0.001, p < 0.001) Right external capsule (p = 0.005, 12 months only) Right superior longitudinal fasciculus (p = 0.003, p < 0.001)
Postoperative increases in MD following right ATLR	Corpus callosum, genu (p = 0.036, 3–4 months only) Corpus callosum, body (p = 0.036, 3–4 months only) Right fornix (cres) (p = 0.002, 12 months only) Right anterior limbic of internal capsule (p = 0.021, 3–4 months only) Right retrolenticular portion of internal capsule (p = 0.009, p < 0.001) Right anterior corona radiata (p = 0.016, p = 0.001) Right superior corona radiata (p = 0.034, 3–4 months only) Right posterior thalamic radiation/optic radiation (p = 0.016) Right inferior fronto-occipital fasciculus (p = 0.005, p < 0.001) Right inferior longitudinal fasciculus (p = 0.006, p < 0.001) Right external capsule (p = 0.005, p < 0.001) Right superior longitudinal fasciculus (p = 0.034, 3–4 months only) Right uncinate fasciculus (p = 0.005, p < 0.001)

**Table 5:** Summary of magnitude of longitudinal changes in diffusion parameters in the clusters of significantly altered FA in patients with TLE. Values are quoted as mean (standard deviation). MD, AD, RD and given in units of mm^2^ s^− 1^ × 10^− 3^. The % change is the change from baseline to 12 months post surgery.

Parameter	Baseline	3–4 months	12 months	% change (p-value)
*Left TLE: cluster of decreased FA*				
FA	0.424 (0.030)	0.384 (0.032)	0.381 (0.031)	− 10.1% (p < 0.001)
MD	0.840 (0.049)	0.882 (0.057)	0.889 (0.054)	+ 5.8% (p < 0.001)
AD	1.254 (0.045)	1.258 (0.045)	1.263 (0.043)	+ 0.7% (p = 0.204)
RD	0.632 (0.054)	0.695 (0.066)	0.701 (0.062)	+ 10.9% (p < 0.001)

*Left TLE: cluster of increased FA*				
FA	0.438 (0.024)	0.467 (0.022)	0.476 (0.021)	+ 8.5% (p < 0.001)
MD	0.755 (0.032)	0.759 (0.033)	0.757 (0.031)	+ 0.3% (p = 0.474)
AD	1.143 (0.035)	1.184 (0.043)	1.193 (0.039)	+ 4.3% (p < 0.001)
RD	0.561 (0.034)	0.547 (0.033)	0.540 (0.032)	− 3.8% (< 0.001)

*Right TLE: cluster of decreased FA*				
FA	0.441 (0.023)	0.411 (0.024)	0.408 (0.024)	− 7.5% (p < 0.001)
MD	0.795 (0.028)	0.822 (0.034)	0.821 (0.037)	+ 3.3% (p < 0.001)
AD	1.207 (0.026)	1.206 (0.029)	1.200 (0.029)	− 0.6% (p = 0.168)
RD	0.589 (0.035)	0.631 (0.041)	0.632 (0.043)	+ 7.3% (p < 0.001)

*Right TLE: cluster of increase FA*				
FA	0.469 (0.028)	0.505 (0.028)	0.517 (0.021)	+ 10.3% (p < 0.001)
MD	0.725 (0.019)	0.729 (0.020)	0.728 (0.019)	+ 0.5% (p = 0.407)
AD	1.136 (0.049)	1.187 (0.043)	1.201 (0.046)	+ 5.8% (p < 0.001)
RD	0.519 (0.021)	0.500 (0.024)	0.492 (0.016)	− 5.3% (p < 0.001)

## Footnotes

1 AD = axial diffusivity, ATLR = anterior temporal lobe resection, DTI = diffusion tensor imaging, EEG = electroencephalography, FA = fractional anisotropy, IFOF = inferior fronto-occipital fasciculus, ILF = inferior longitudinal fasciculus, MD = mean diffusivity, PHC = parahippocampal cingulum, PTR/OR = posterior thalamic radiation/optic radiation, RD = radial diffusivity, SLF = superior longitudinal fasciculus, TBSS = tract-based spatial statistics, TFCE = threshold-free cluster enhancement, TLE = temporal lobe epilepsy, UF = uncinate fasciculus.
