# Supplementary material for: Progressive white matter changes following anterior temporal lobe resection for epilepsy
Source: Neuroimage Clin. 2013 Dec 14;4:190–200. doi: 10.1016/j.nicl.2013.12.004 (PMC3879413; doi:10.1016/j.nicl.2013.12.004)
Supplement: Supplementary file 1 — Supplementary tables. [file mmc1.docx]

Supplementary Table 1. Summary of baseline diffusion changes in whole-brain analysis of left TLE patients in comparison to controls. The p-values are the maximum difference observed in each structure.

| Baseline decreases in FA | Corpus callosum, genu (p=0.034) Corpus callosum, body (p=0.035) Corpus callosum, splenium (p=0.049) Left fornix (cres) (p=0.035) Left anterior limb of internal capsule (p=0.033) Left posterior limb of internal capsule (p=0.043) Left retrolenticular part of internal capsule (p=0.035) Left anterior corona radiata (p=0.033) Right anterior corona radiata (p=0.036) Left superior corona radiata (p=0.034) Right superior corona radiata (p=0.036) Left posterior corona radiata (p=0.048) Left posterior thalamic radiation (p=0.037) Left inferior fronto-occipital fasciculus (p=0.033) Left inferior longitudinal fasciculus (p=0.034) Left external capsule (p=0.033) Left superior longitudinal fasciculus (p=0.034) Left uncinate fasciculus (p=0.034)  Left corticospinal tract (p=0.041) Left cerebral peduncle (p=0.042) Right cerebral peduncle (p=0.045) Left superior cerebellar peduncle (p=0.042) Right superior cerebellar peduncle (p=0.042) Middle cerebellar peduncle (p=0.041)  Left inferior cerebellar peduncle (p=0.045) Left medial lemniscus (p=0.041) Right medial lemniscus (p=0.045) |
| --- | --- |
| Baseline increases in MD | Corpus callosum, genu (p=0.040) Left fornix (cres) (p=0.043) Left anterior limb of internal capsule (p=0.038) Left anterior corona radiata (p=0.038) Left inferior fronto-occipital fasciculus (p=0.038) Left inferior longitudinal fasciculus (p=0.033) Left external capsule (p=0.038) Left uncinate fasciculus (p=0.038) |

Supplementary Table 2. Summary of baseline diffusion changes in whole-brain analysis of right TLE patients in comparison to controls. The p-values are the maximum difference observed in each structure.

| Baseline decreases in FA | Corpus callosum, genu (p=0.003) Corpus callosum, body (p=0.003) Corpus callosum, splenium (p=0.006) Column/body of fornix (p=0.012) Left fornix (cres) (p=0.001) Right fornix (cres) (p=0.003) Left anterior limb of internal capsule (p=0.022) Right anterior limb of internal capsule (p=0.005) Left posterior limb of internal capsule (p=0.022) Right posterior limb of internal capsule (p=0.005) Left retrolenticular portion of internal capsule (p=0.019) Right retrolenticular portion of internal capsule (p=0.004) Left anterior corona radiata (p=0.007) Right anterior corona radiata (p=0.003) Left superior corona radiata (p=0.009) Right superior corona radiata (p=0.004) Right posterior corona radiata (p=0.006) Right posterior thalamic radiation/optic radiation (p=0.023) Left inferior fronto-occipital fasciculus (p=0.007) Right inferior fronto-occipital fasciculus (p=0.003) Left inferior longitudinal fasciculus (p=0.013) Right inferior longitudinal fasciculus (p=0.003) Left external capsule (p=0.010) Right external capsule (p=0.003)  Left cingulate gyrus (p=0.005) Right cingulate gyrus (p=0.005) Right parahippocampal cingulum (p=0.040) Right superior longitudinal fasciculus (p=0.017) Right superior fronto-occipital fasciculus (p=0.012) Left uncinate fasciculus (p=0.010)  Right uncinate fasciculus (p=0.003)  Left corticospinal tract (p=0.012) Right corticospinal tract (p=0.032) Left cerebral peduncle (p=0.014) Right cerebral peduncle (p=0.005) Left superior cerebellar peduncle (p=0.012) Right superior cerebellar peduncle (p=0.011) Middle cerebellar peduncle (p=0.012) Left inferior cerebellar peduncle (p=0.012) Right inferior cerebellar peduncle (p=0.012) Left medial lemniscus (p=0.012) Right medial lemniscus (p=0.012) |
| --- | --- |
| Baseline increases in MD | Corpus callosum, genu (p=0.008) Corpus callosum, body (p=0.012) Right fornix (cres) (p=0.005) Left anterior limb of internal capsule (p=0.033) Right anterior limb of internal capsule (p=0.023) Right retrolenticular portion of internal capsule (p=0.006) Left anterior corona radiata (p=0.016) Right anterior corona radiata (p=0.007) Left superior corona radiata (p=0.026) Right superior corona radiata (p=0.029) Right posterior thalamic radiation/optic radiation (p=0.029) Left inferior fronto-occipital fasciculus (p=0.016) Right inferior fronto-occipital fasciculus (p=0.002) Left inferior longitudinal fasciculus (p=0.033) Right inferior longitudinal fasciculus (p=0.002) Left external capsule (p=0.032) Right external capsule (p=0.002) Left cingulate gyrus (p=0.048) Right parahippocampal cingulum (p=0.046) Right superior longitudinal fasciculus (p=0.033) Left uncinate fasciculus (p=0.033)  Right uncinate fasciculus (p=0.002) |
